# Optical Coherence Tomography and Autofluorescence Imaging of Human Tonsil

**DOI:** 10.1371/journal.pone.0115889

**Published:** 2014-12-26

**Authors:** Hamid Pahlevaninezhad, Anthony M. D. Lee, Miriam Rosin, Ivan Sun, Lewei Zhang, Mehrnoush Hakimi, Calum MacAulay, Pierre M. Lane

**Affiliations:** 1 Department of Integrative Oncology, British Columbia Cancer Research Center, Vancouver, British Columbia, Canada; 2 Department of Dentistry, University of British Columbia, Vancouver, British Columbia, Canada; University of Pennsylvania, United States of America

## Abstract

For the first time, we present co-registered autofluorescence imaging and optical coherence tomography (AF/OCT) of excised human palatine tonsils to evaluate the capabilities of OCT to visualize tonsil tissue components. Despite limited penetration depth, OCT can provide detailed structural information about tonsil tissue with much higher resolution than that of computed tomography, magnetic resonance imaging, and Ultrasound. Different tonsil tissue components such as epithelium, dense connective tissue, lymphoid nodules, and crypts can be visualized by OCT. The co-registered AF imaging can provide matching biochemical information. AF/OCT scans may provide a non-invasive tool for detecting tonsillar cancers and for studying the natural history of their development.

## Introduction

Human tonsils are lymphoid tissues that often represent the first line of biological defence against foreign pathogens [1]. Stratified squamous epithelium covers the paired palatine tonsils from the pharyngeal side and extends deep into tonsillar crypts, increasing greatly the tonsil's contact surface to the outside environment for early exposure of immune system cells to infectious organisms. The netlike tonsillar epithelium structure facilitates the transport of antigens in order to make tonsils more sensitive to external pathogens [2]. However, these epithelial disruptions cause the basement membrane to be exposed and more susceptible to infections, particularly in crypts. There are reports suggesting that most tonsillar cancers associated with human papillomavirus (HPV) infection could begin in the tonsillar crypts [3]–[6], obscured from the naked eye. Unlike in the cervix, the tonsillar epithelium covers a non-flat mucosal surface with several invaginations. These particular structural features render Pap-test impractical for detecting tonsillar pre-cancer or cancer [7], [8].

Our understanding of the natural history of HPV infection in cancer pathogenesis is greatly limited by our inability to visualize such change without biopsy [2], [9], [10]. Therefore, an imaging system capable of providing tonsillar tissue morphology would be of great value to study the processes involved in early tonsillar cancers and other tonsillar diseases such as recurrent tonsillitis. The most common imaging modalities for tonsil imaging include computed tomography/positron emission tomography (CT/PET) [11]–[17] and magnetic resonance imaging (MRI) [18]–[22]. However, owing to their limited resolution, these imaging modalities show low sensitivity in the detection of primary lesions [17], [18], [22]–[26]. Ultrasound (US) imaging has also been utilized for tonsil imaging [27]–[30] with an improved rate of detection of unknown primary compared to MRI and PET/CT [30].

Optical coherence tomography (OCT) is an interferometric technique for obtaining sub-surface tissue morphology, providing images millimetres deep in the tissue with about 10 µm axial resolution [31]–[33], significantly higher (at least an order of magnitude) than other aforementioned tonsillar imaging techniques. OCT is less invasive compared to PET/CT since it employs nonionizing, near-infrared radiation to capture real-time images of tissue morphology. OCT can be used to study high-risk tissue sites without requiring biopsies and tissue removal [34]. Autofluorescence (AF) is the emission of light by endogenous fluorophores in biological structures when illuminated with the appropriate excitation light. In tissue, the most diagnostically useful fluorophores excited by blue excitation light are collagen, elastin, and nicotinamide adenine dinucleotide phosphate [35]. The intensity and spectrum of tissue AF is determined by the quantity of fluorophores *in situ* as well as their biochemical environment. Changes in the intensity and spectral distribution of tissue AF can be used to follow pathological processes such as carcinogenesis. Thus, when used in combination, AF/OCT systems can provide biochemical information co-localized with structural information that could be used to study different disease processes.

In this work, we present experimental results of AF/OCT scans of excised palatine tonsils using a custom benchtop AF/OCT system. To our knowledge, this is the first report on AF/OCT scans for tonsil tissue, presenting a first step toward the possibility of high-resolution imaging of tonsil tissue components.

## Materials and Methods

### Tonsil tissue preparation

Collection of samples for this study was approved by the University of British Columbia and British Columbia Cancer Agency Research Ethics Board. All participants provided written consent approved by the ethics committees. Sixteen palatine tonsils (2 per patient) were obtained during conventional tonsillectomy. Patients had a clinical history of tonsillitis but no evidence of malignancy at that site. The fresh samples were transferred immediately to the lab in Dulbecco's Modified Eagle Medium (DMEM) for imaging after surgery. Eight of these tonsils were placed between two glass slides for AF/OCT scans of the whole tonsils. Due to the limited penetration depth of OCT, tonsillar tissue components located (more than a few millimetres) deep inside the tonsils could not be visualized. To visualise these component by OCT, thin sections (about 3 mm thick) were also cut out from the anterior, middle, and posterior of the remaining 8 tonsils, placed between two glass slides, and imaged with the system. After imaging, samples were fixed with 10% formalin for 24 hours at room temperature, dehydrated with ethanol, cleared with xylene, and embedded in paraffin for histopathology evaluations. 4 µm-thick sections were cut, transferred onto slides, deparaffinised, H&E stained, and scanned on a slide scanner (Panoramic MIDI Scanner, 3DHisttech, Budapest, Hungary).

### AF/OCT system

**Figure 1:**
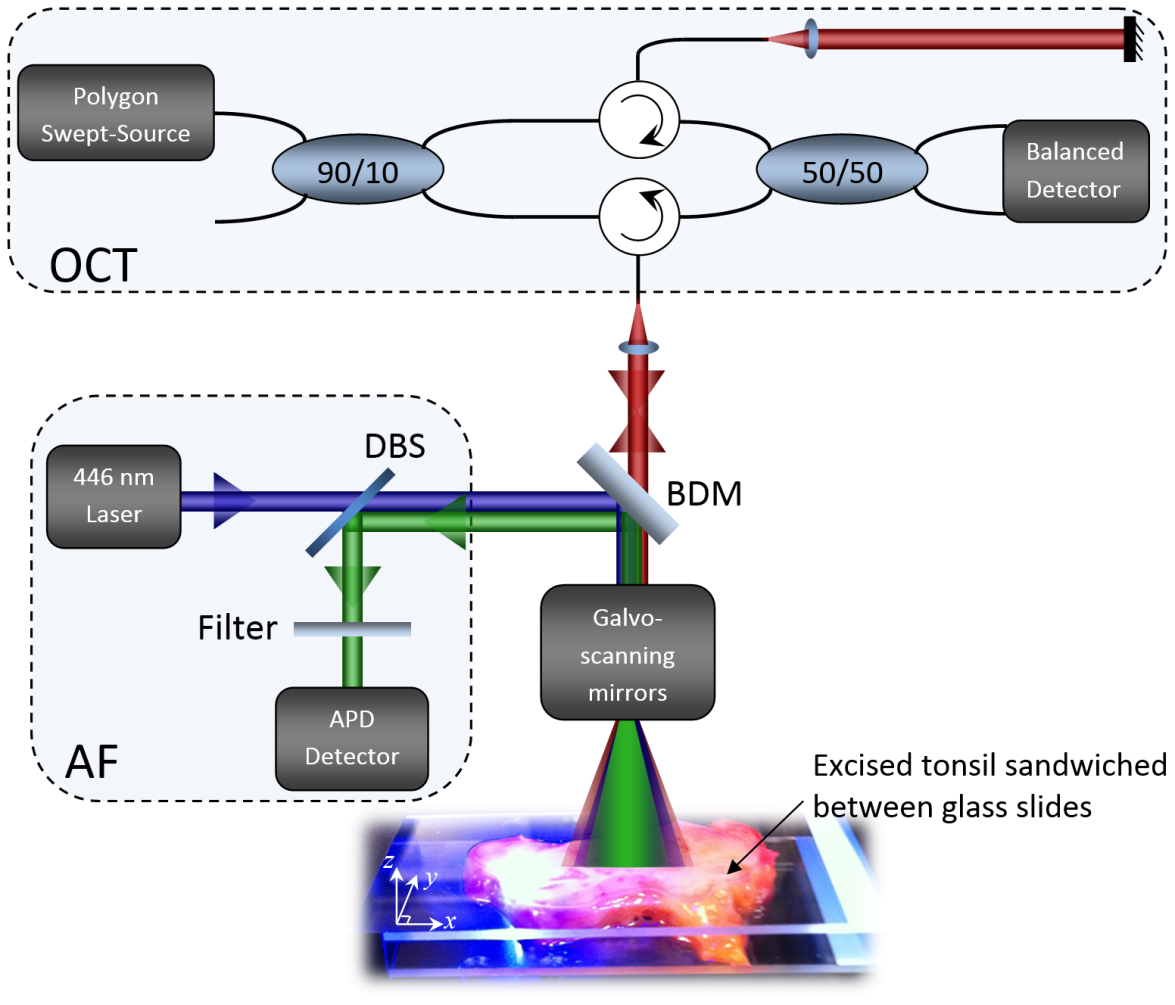
AF/OCT system. AF/OCT system for imaging excised tonsils placed between glass slides, BDM: broadband dielectric mirror, DBS: dichroic beamsplitter, APD: avalanche photodiode.

Co-registered AF/OCT scans were acquired using a custom bench-top dual-modality imaging system illustrated in Fig. 1. The details of the AF/OCT system can be found in our previous publication [36]. The OCT subsystem includes a fiber-based Mach-Zehnder interferometer driven by a 30-mW polygon-scanner-based wavelength-swept laser source with 106.8 nm bandwidth centered at 1321.4 nm with 40-kHz repetition frequency (OCT inset). The interference is detected by a balanced photodetector whose output is fed into one channel of a digitizer card (ATS460, AlazarTech, Pointe-Claire, QC) for signal processing and OCT display. The AF imaging subsystem uses a 446 nm, 40 mW semiconductor laser (CUBE 445-40C, Coherent, Santa Clara, CA, USA) as the excitation source. An objective lens (NA = 0.3) with 40-mm working distance collects the AF emission from the sample and another lens is used to focus the collected emission onto an APD-based detector (C5460, Hamamatsu, Japan). A dichroic beamsplitter (DBS) separates back-scattered AF blue excitation light from AF emission as shown in Fig. 1 (AF inset). The OCT and AF light beams are combined and separated by a backside-polished broadband dielectric mirror (BB1-E02P, ThorLabs, Newton, NJ, USA) in free space. A Galvo-scanning mirror (GVS002, ThorLabs, Newton, NJ, USA) provides a two-dimensional (2-D) raster scan of both the AF and OCT beams on the sample. The AF and OCT channels are recorded simultaneously on the same high speed digitizer ensuring their coregistration.

The structural volumetric images of the samples were acquired by 2-D raster scans of the OCT beam on the samples with the depth information. The 3-D structural images were constructed from individual OCT frames (B-scans, parallel to *x*-*z* plane in Fig. 1) and sectioned into the en-face planes (parallel to *x*-*y* plane in Fig. 1) perpendicular to the excitation light. En-face OCT sections were compared to the AF images and the histology results. Also OCT B-scans were compared to the corresponding histology results. AF data points were averaged over the length of OCT A-lines (OCT depth information) as no depth information is encoded in the AF data stream.

## Results and Discussion

**Figure 2:**
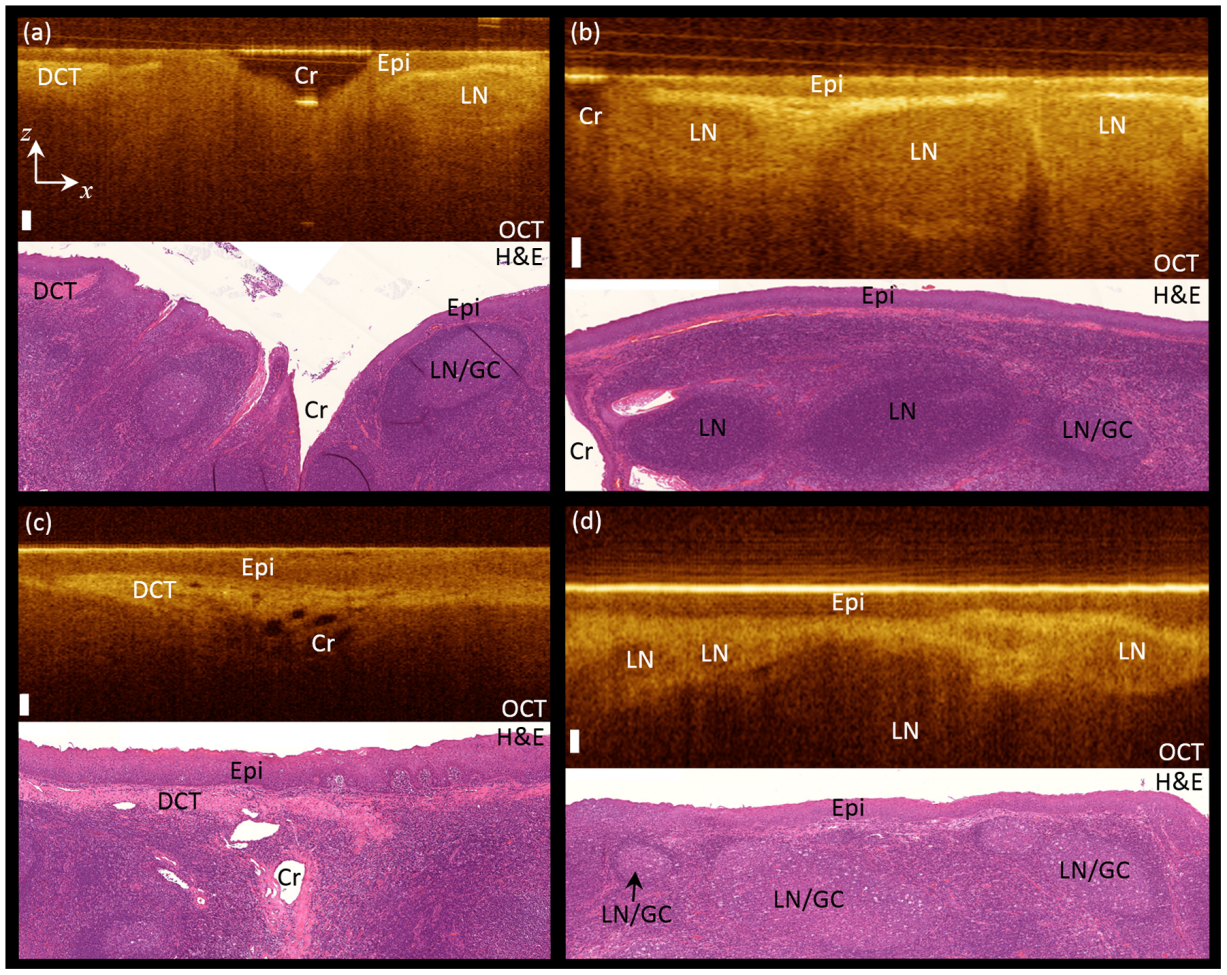
OCT vs. histology. Comparing OCT B-scans with histopathology results, DCT: dense connective tissue, Epi: epithelium, Cr: crypt, LN: lymphoid nodules, and GC: germinal center. White bar is 100 µm.

**Figure 3:**
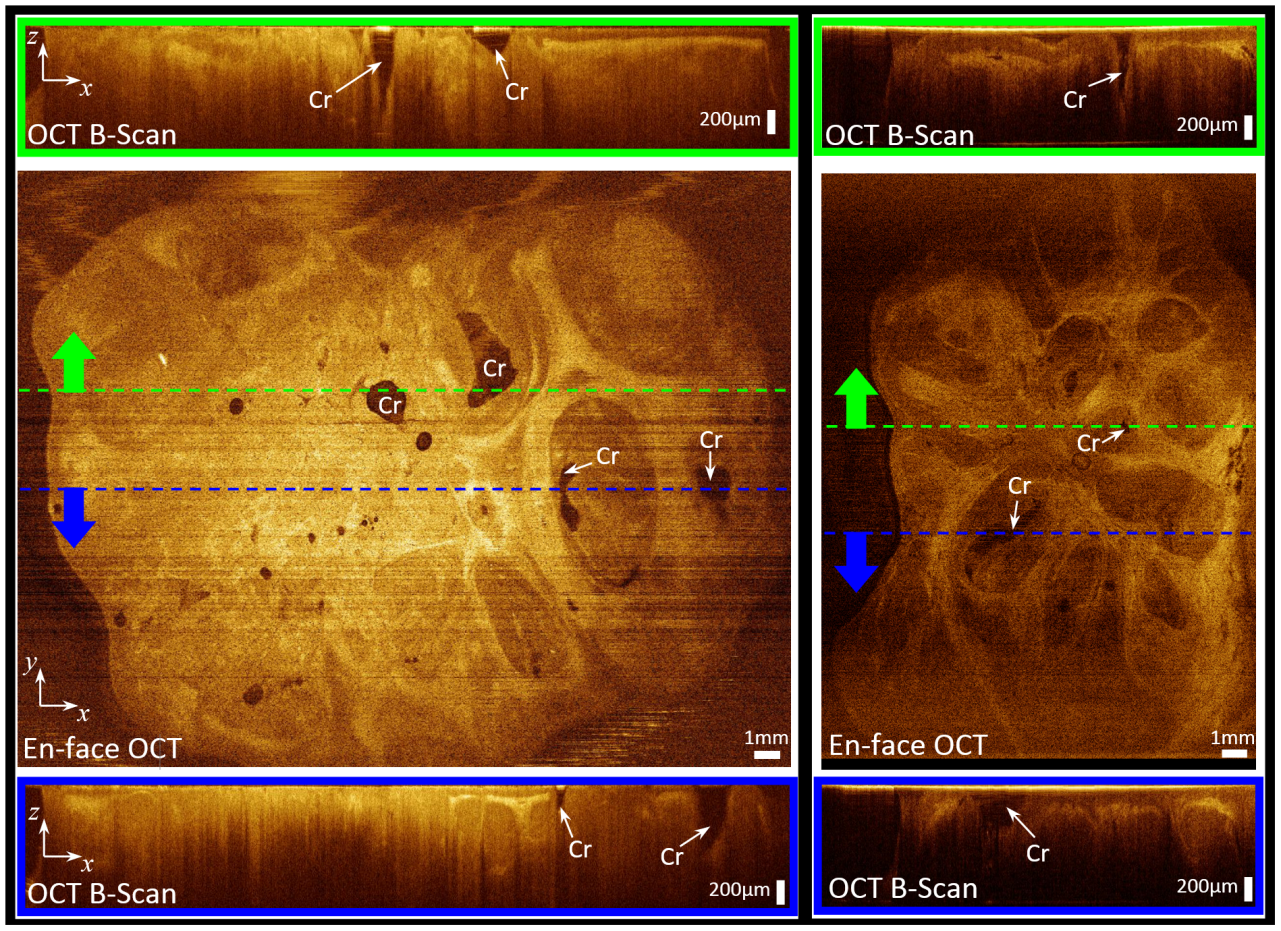
Visualizing crypts by OCT. Middle row are enface OCT sections, top and bottom rows are OCT B-scans corresponding to the green and blue dashed lines, respectively; Cr: crypt.

Fig. 2 compares the OCT B-scans with the histopathology results. Different tonsil tissue components can be clearly identified in the OCT scans, including epithelium (Epi), dense connective tissue (DCT), crypts (Cr), and lymph nodules/germinal centers (LN/GC), a feature unavailable to the alternative imaging techniques such as CT/PET, MRI, and US. Dense connective tissue appears bright and crypts are dark in the OCT scans. Lymphoid nodules appear darker than neighbouring tissue with distinct borders in the OCT scans. The OCT ability to visualize tonsillar tissue components holds promise for using OCT to monitor the detailed structural changes in tonsil tissue due to different disease processes. In particular, imaging inside the crypts could be potentially used for primary lesion detection inside the crypts. Fig. 3 illustrates the capability of OCT in visualizing tonsillar crypts in two tonsil samples. Two representative en-face OCT sections of these two samples (the ones that show the crypts more clearly) are chosen to be illustrated in the middle panel of Fig. 3. The OCT B-scans (framed in green and blue), illustrated in the top and bottom panels, corresponding to the dashed green and blue lines in the enface images clearly show the crypts' shape and structure deep inside the tonsillar tissue.

**Figure 4:**
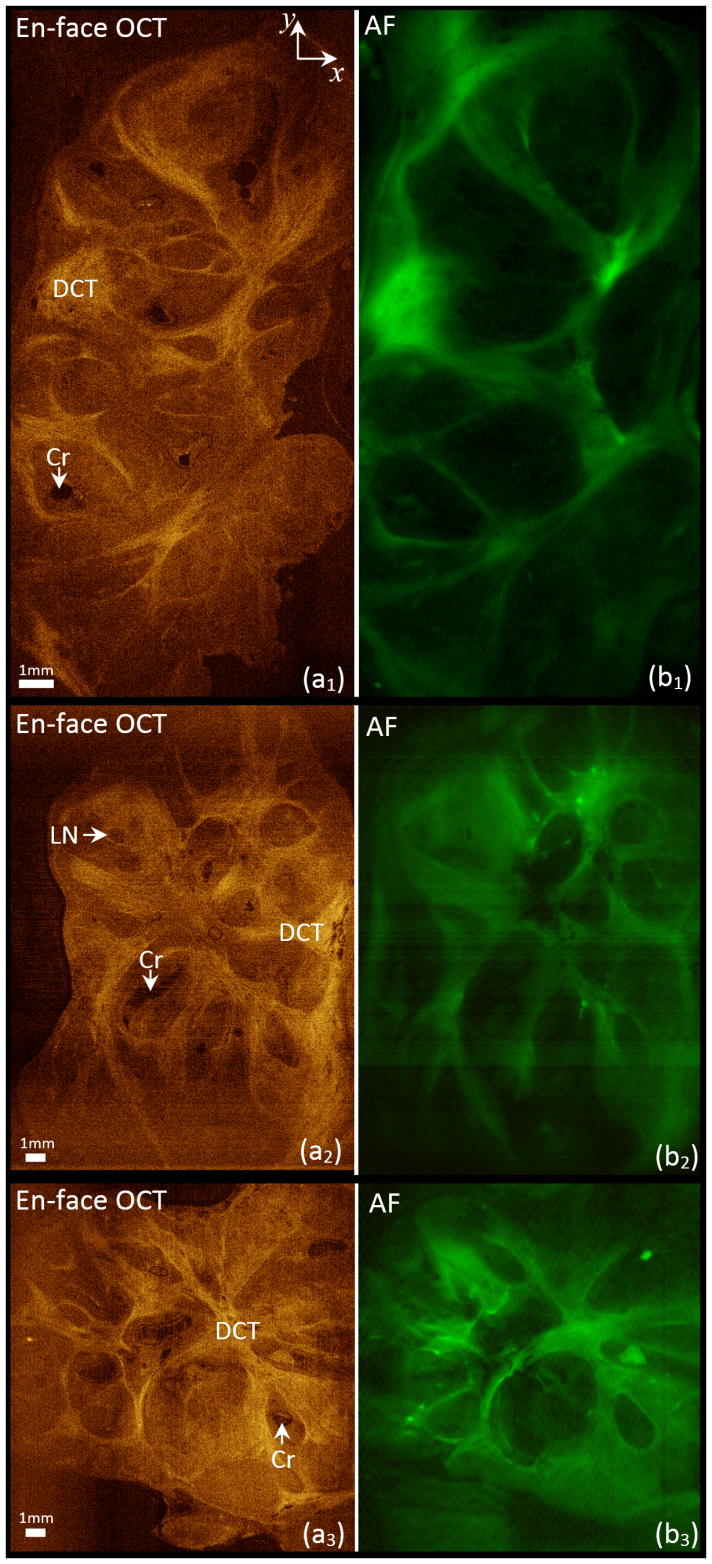
AF/OCT scans of tonsils. Imaging results obtained from AF/OCT scans of three tonsils samples, including representative enface OCT (a_1-3_), green AF images (b_1-3_); Cr: crypt, DCT: dense connective tissue, LN: lymphoid nodules.

Fig. 4 shows imaging results from three tonsil samples including enface OCT (a_1-3_) and AF images (b_1-3_). For each sample, the 3-D structural image constructed from OCT B-scans (S1-S3 Files include full B-scans for the three samples) is sectioned perpendicular to the excitation light (parallel to *x*-*y* plane in Fig. 1) in order to generate enface OCT sections. The first column of Fig. 4 shows three representative enface OCT sections for the three samples (S4-S6 Files include full enface OCT sections for the three samples). Different tonsil tissue components including dense connective tissue (DCT), lymphoid nodules (LN), and crypts (Cr) can be identified in the OCT B-scan frames and enface OCT sections. The second column of Fig. 4 shows the corresponding AF images. Dense connective tissue areas are distinctive features in the AF image. They appear bright as they create strong AF signal due to the high concentration of collagen fibers. The AF signal is small in the crypts as expected. The AF image can visualise biochemical properties of tonsillar tissue components such as their relative concentration of fluorescing fibers.

The dense connective tissue provides high signal intensity in both the AF and OCT images of Fig. 4. The cross-links of the collagen fibers provide high AF contrast while the refractive-index differences of the fibers provide high backscatter contrast. Tissue components don't always provide both types of contrast simultaneously. For instance, in airway tissue, cartilage usually appears dark in OCT while provides very high fluorescent signal [35]. Importantly, the AF signal intensity depends on the density of cross-links that often correlates with dysplastic progression. Therefore, AF imaging may be useful in studying the development of tonsillar cancer.

**Figure 5:**
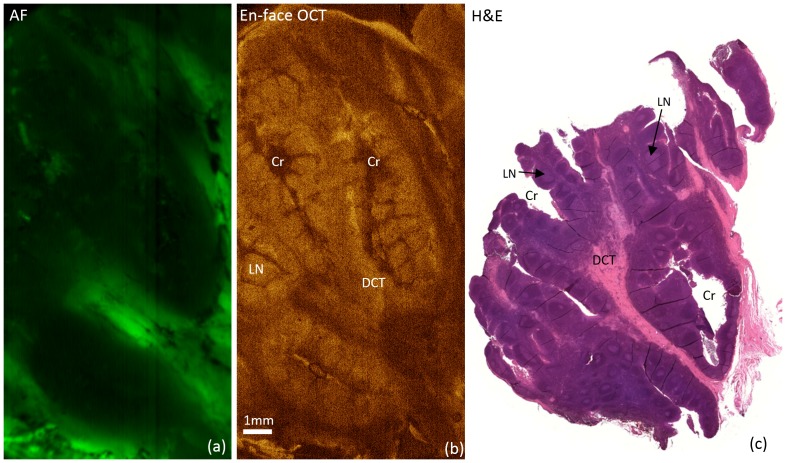
AF/OCT scan of thin tonsil section. (a) AF image and (b) representative enface OCT section from a thin section cut from a tonsil sample along with the histopathology result (c) obtained from a 4 µm thick section cut from the same sample Cr: crypt, LN: lymphoid nodules, and DCT: dense connective tissue.

Fig. 5 shows imaging results from one of the thin sections (3 mm thick) cut from tonsil samples. The enface OCT section clearly visualizes tonsil tissue components such as dense connective tissue (DCT), lymphoid nodules (LN), and crypts (Cr) (S7 File includes full enface OCT sections of this sample). Dense connective tissue areas are noticeable in the AF images. Histopathology results from a 4 µm-thick section cut from the same sample are illustrated in Fig. 5(c).

Results show that tonsillar tissue components can be identified by OCT with the resolutions that cannot be achieved by other alternative imaging techniques such as US, CT/PET, and MRI. Given this capability, we expect that OCT can be a useful technique in studying the processes involved in early tonsillar cancer. For instance, this method could potentially provide insight as to whether HPV infection begins in the tonsillar crypts or not, provided that primary lesions inside the crypts can be visualized by OCT.

This work was intended to investigate the capabilities of AF/OCT for tonsil imaging by comparing imaging results with histopathology. The imaging results were obtained from *ex vivo* samples using the bench-top AF/OCT system since the comparison between the imaging results with histopathology can be achieved easier with *ex vivo* samples. However, using a fiber-based AF/OCT system [37], the same technique can be extended to imaging tonsils *in vivo*.

## Conclusions

This work presents experimental results from AF/OCT scans of excised palatine tonsils using a custom AF/OCT system. The imaging results are compared to the histology results to evaluate the AF/OCT scan capabilities. Different tonsillar tissue components can be clearly visualised by OCT and AF imaging provides biochemical properties of tonsil tissue. To our knowledge, this is the first report on AF/OCT scans of tonsil tissue, presenting a first step toward high-resolution imaging of tonsil tissue components. This method may be useful to study how different tonillar disease processes change the biochemical and structural properties of tonsillar tissue.

## Supporting Information

S1 File **OCT B-scan video.** This movie includes all OCT B-scan frames for the sample illustrated in Fig. 4(a_1_).
(AVI)

S2 File **OCT B-scan video.** This movie includes all OCT B-scan frames for the sample illustrated in Fig. 4(a_2_).
(AVI)

S3 File **OCT B-scan video.** This movie includes all OCT B-scan frames for the sample illustrated in Fig. 4(a_3_).
(AVI)

S4 File **Enface OCT video.** This movie includes all enface OCT frames for the sample illustrated in Fig. 4(a_1_).
(AVI)

S5 File **Enface OCT video.** This movie includes all enface OCT frames for the sample illustrated in Fig. 4(a_2_).
(AVI)

S6 File **Enface OCT video.** This movie includes all enface OCT frames for the sample illustrated in Fig. 4(a_3_).
(AVI)

S7 File **Enface OCT video.** This movie includes all enface OCT frames for the sample illustrated in Fig. 5.
(AVI)
